# Extracellular caspase-1: a critical inducer and a therapeutic target of lung injury in gut ischemia-reperfusion

**DOI:** 10.3389/fimmu.2026.1811868

**Published:** 2026-04-15

**Authors:** Kouhei Ishikawa, Gaifeng Ma, Alok Jha, Atsushi Murao, Ping Wang, Monowar Aziz

**Affiliations:** 1 Center for Immunology and Inflammation, The Feinstein Institutes for Medical Research, Manhasset, NY, United States; 2 Departments of Surgery and Molecular Medicine, Zucker School of Medicine at Hofstra/Northwell, Manhasset, NY, United States

**Keywords:** ALI, DAMPs, extracellular caspase-1, GSDMD, gut I/R, TLR4

## Abstract

Caspase-1 is known to function intracellularly. However, whether caspase-1 can be released extracellularly and if so, the mechanisms of its release and action remain unknown. Here, we identify that cleaved caspase-1 (p20), which we named extracellular caspase-1 (eCasp-1), is released from immune cells in association with gasdermin D (GSDMD) pores formation. We identified significantly elevated eCasp-1 levels in the blood of critically ill surgical ICU patients, and in the blood and peritoneal fluid of gut ischemia-reperfusion (I/R) injury mice. *In vitro*, hypoxia-reoxygenation promoted GSDMD-dependent eCasp-1 release, and pharmacological inhibition of GSDMD reduced this release, supporting a contributing role of GSDMD-mediated membrane permeabilization in eCasp-1 release. Gut I/R demonstrated robust GSDMD-dependent release of eCasp-1. Functionally, eCasp-1 engaged TLR4 on macrophages, eliciting robust inflammatory cytokine release and organ injury. Importantly, we developed a novel peptide, C16, designed to specifically inhibit the eCasp-1-TLR4 interaction. In gut I/R, C16 administration exerted therapeutic benefits, markedly reducing systemic inflammation, attenuating acute lung injury (ALI), and significantly improving the survival. Our findings identify eCasp-1 as a new alarmin, that contributes to ALI through TLR4 signaling, with GSDMD-dependent processes contributing to its extracellular release. Targeting eCasp-1 with C16 offers a promising therapeutic strategy against ALI in acute inflammation.

## Introduction

Caspase-1, an intracellular cysteine protease, converts pro-IL-1β and pro-IL-18 into their mature forms and serves as a principal effector of inflammasome activation (1–3). Predominantly expressed in myeloid cells, caspase-1 exists intracellularly as an inactive proenzyme (45-kDa) and undergoes self-cleavage upon inflammasome assembly to generate the active heterotetramer (p20/p10) (3, 4). Beyond cytokine maturation, cleaved caspase-1 mediates the proteolytic activation of gasdermin D (GSDMD), thereby driving pyroptosis and amplifying inflammation (5–7). However, whether cleaved caspase-1 itself is released into the extracellular milieu and what function it may exert outside the cells remains unexplored.

GSDMD-mediated pore formation has emerged as a pivotal mechanism for the extracellular release of inflammatory mediators. Upon cleavage by inflammasome-activated caspase-1 or caspase-11, the N-terminal fragment of GSDMD (GSDMD-NT) oligomerizes to form nanoscale pores in the plasma membrane, facilitating the release of IL-1β, IL-18, ATP, and various damage-associated molecular patterns (DAMPs), and inducing pyroptotic cell death (2, 8). Interestingly, recent studies have demonstrated that cells can survive following GSDMD pore formation, entering a “hyperactivated state” in which low-molecular-weight inflammatory mediators are selectively secreted without cell lysis. Based on these insights, we hypothesized that cleaved caspase-1 (p20), in association with GSDMD-dependent membrane processes, including pore formation, acts as a novel DAMP.

Systemic inflammatory response syndrome (SIRS) arises from severe infection or tissue injury. It is a central mechanism in life-threatening conditions like gut ischemia-reperfusion (I/R), frequently leading to multi-organ dysfunction and death (9, 10). Gut I/R, characterized by transient intestinal ischemia followed by reperfusion, triggers excessive inflammation and tissue damage and occurs in diverse clinical contexts including surgery, sepsis, trauma, and post-cardiac arrest resuscitation. Disruption of the intestinal epithelial barrier and bacterial translocation are considered key drivers of SIRS in this setting (9–11). Importantly, gut I/R frequently give rise to acute lung injury (ALI), a major secondary complication that exacerbates systemic inflammation and markedly increases mortality (12, 13). ALI is characterized by diffuse alveolar damage, disruption of the alveolar capillary barrier, and massive neutrophil infiltration, resulting in pulmonary edema, hypoxemia, and impaired gas exchange. These pulmonary manifestations reflect a distant organ response driven by the spillover of inflammatory mediators, including DAMPs, from the primary insult, further amplifying the vicious cycle of inflammation and multi organ failure (12, 13). Despite the intricate pathophysiology of these inflammatory disorders, FDA-approved drugs are largely absent, with only supportive care currently available (14, 15). This highlights an urgent unmet need for the discovery of novel and effective therapeutic candidates.

In this study, we aim to investigate whether eCasp-1 is released during inflammation and functions as a new DAMP. We will further elucidate its mechanism of release from inflamed cells and its role in exacerbating inflammation, tissue injury, and cell death. Ultimately, we aim to discover and develop a novel therapeutic molecule to target eCasp-1, thereby mitigating inflammation and ALI, and improving survival in acute inflammatory disease conditions.

## Materials and methods

### Human samples

Peripheral blood samples (5 mL each) were collected from deidentified healthy volunteers without specific underlying conditions and from deidentified patients admitted to the surgical intensive care unit (SICU). All participants provided written informed consent, and the study protocol was approved by the Northwell Health Institutional Review Board (IRB protocol # 22-0458). The whole blood was obtained by venipuncture and collected into EDTA-containing tubes. The samples were centrifuged at 1,600 × g for 10 min to separate plasma, which was aliquoted and stored at -80 °C for further analysis.

### Animal experiments

Adult male wild-type (WT) C57BL/6 mice (8–12 weeks, 20–25 g; Charles River Laboratories, Wilmington, MA) and TLR4-knockout (TLR4^-/-^) mice (bred in-house from breeders provided by Dr. Kevin Tracey, The Feinstein Institutes for Medical Research, Manhasset, NY) were used. Mice were housed under standard conditions with a 12-h light-dark cycle, provided standard chow, and acclimated for 5–7 days. Given the effects of sexual dimorphism on clinical, pathophysiological, and immunological outcomes, we used a single gender male mouse (16). All procedures were approved by the Institutional Animal Care and Use Committee of the Feinstein Institutes for Medical Research (protocol numbers: 24-0620) and conducted in accordance with the National Institutes of Health and the Guide for the Care and Use of Laboratory Animals.

### Gut I/R model

Gut I/R was performed as previously described (17). Briefly, mice were anesthetized with 2-4% inhalational isoflurane and positioned supine on a heated surgical platform. After confirming anesthesia, a 1.5 cm midline laparotomy was performed, and the superior mesenteric artery (SMA) was occluded for 60 min using atraumatic vascular clamps (Item No. 18055-04, Fine Science Tools, Foster City, CA). Clamps were then removed to allow reperfusion. The abdomen was closed, and mice received subcutaneous normal saline (0.5 mL) for fluid support and 0.1 µg/g body weight (BW) buprenorphine for analgesia. Anesthesia was reversed, and 1.0% lidocaine was applied topically to the incision site. Sham-operated mice underwent anesthesia and laparotomy without SMA occlusion. After 2 or 4 h of reperfusion, depending on the experiment, mice were euthanized, and blood and peritoneal cavity fluid were collected. Blood samples were centrifuged at 1,000 × g for 15 min to obtain plasma, while peritoneal cavity fluid was centrifuged at 400 × g for 10 min to collect supernatants. All samples were stored at -80 °C for further analysis.

### Isolation of peritoneal macrophages

Primary peritoneal macrophages were isolated from WT or TLR4^-/-^ mice. Mice were euthanized by CO_2_ asphyxiation, and the peritoneal cavity was lavage with ice-cold PBS containing 2% fetal bovine serum (FBS; Invitrogen). Lavage fluid was collected using a 10 mL syringe, and cells were pelleted by centrifugation at 400 × g for 10 min. The cell pellet was resuspended in RPMI 1640 medium (Invitrogen) supplemented with 25 mM HEPES, 2 mM glutamine, 10% FBS, and 100 IU/mL penicillin. Cell counts were determined by hemocytometer. Approximately 1 × 10^5^ cell were seeded into 96-well or 1 × 10^6^ cell were seeded into 24-well flat-bottom culture plates and incubated at 37 °C for 4 h to promote adhesion. Nonadherent cells were removed by washing twice with warm PBS. Adherent cells, consisting of >90% macrophages, were further cultured overnight at 37 °C in 5% CO_2_. Prior to the experimental treatment, the cells were washed with warm PBS to remove any remaining non-adherent cells, and the culture medium was changed to Opti-MEM medium (Invitrogen).

### Culture of intestinal organoids

Isolation and culture of intestinal crypts and 3D organoids were performed as previously described (18). Briefly, small intestines from WT mice were harvested, cut into ~5 mm fragments, and incubated in ice-cold PBS containing 2.5 mM EDTA and 0.1% bovine serum albumin (BSA; Fisher BioReagents, Pittsburgh, PA) for 30 min. Following removal of the supernatant, tissue fragments were vigorously pipetted in PBS with 0.1% BSA to release crypts. The suspension was filtered through a 70 μm cell strainer to remove villi and debris. Growth factor-reduced Matrigel (Corning, Glendale, AL) for the 3D unit basement was used with murine IntestiCult Organoid Growth Medium (STEMCELL Technologies, Vancouver, BC, Canada) according to the manufacturer’s instructions. Crypts were resuspended in an appropriate volume of the pre-mixed Matrigel and IntestiCult, yielding approximately 10,000 to 15,000 crypts/mL. Aliquots of 70 μL were plated into the center of 24-well flat-bottom culture plates and allowed to polymerize at 37 °C for 10 min. Wells were then overlaid with additional IntestiCult medium, which was refreshed every 3 days. Organoids were maintained at 37 °C in a humidified incubator with 5% CO_2_ and used for experiments on day 5 of culture. All analyses were conducted using primary (passage 0) organoids. Prior to the experimental treatment, the culture medium was changed to Opti-MEM medium.

### Recombinant proteins and peptides

Recombinant mouse caspase-1 (p20) (eCasp-1; ≥95% purity) was obtained from Vector Laboratories (Cat. No. LS-G14039, Newark, CA), stored as a lyophilized powder, and reconstituted in phosphate-buffered saline (PBS; Invitrogen, Waltham, MA). Recombinant mouse TLR4 (rmTLR4; ≥90% purity) was purchased from R&D Systems (Cat. No. 9149-TR-050, Minneapolis, MN), supplied in carrier-free form, and reconstituted in sterile PBS according to the manufacturer’s instructions. These aliquots were stored at -80 °C until use. The compound 16 (C16) peptide (sequence: YNYIQTITVNDLQFLR), a 16-amino-acid peptide designed in our laboratory based on the binding site of TLR4 for eCasp-1 (p20), was synthesized by GenScript Biotech (Piscataway, NJ) at ≥95% purity. Both peptides were dissolved in DMSO at 20 mg/mL, diluted with PBS to 1 mg/mL, and stored at -20 °C until use.

### *In vitro* experiments

#### Disulfiram

The GSDMD inhibitor disulfiram (Cat. No. D2950000, Sigma-Aldrich, St. Louis, MO) was dissolved in sterile corn oil and used at 5 μM. The disulfiram preparation, including dissolution and its dosage, were determined based on previous studies (19, 20). Peritoneal macrophages were isolated from WT mice and seeded into 96-well. Cells were treated with PBS or pre-treated with disulfiram for 1 h. For inflammasome activation, cells were stimulated with lipopolysaccharide (LPS) (100 ng/mL) for 3 h, followed by nigericin (10 μM) for an additional 1 h. In a separate experiment, peritoneal macrophages and intestinal organoids were seeded into 24-well plates, pre-treated with PBS or disulfiram (5 μM) for 1 h, and then placed in a sealed hypoxic chamber containing 1% O_2_, 5% CO_2_, and 94% N_2_ at 37 °C for 6 h. Cells were then returned to normoxic conditions (37 °C, 5% CO_2_) for 16 h. After treatments, cell culture supernatants were collected, centrifuged at 400 × g for 10 min, and stored at -20 °C for further analyses.

#### eCasp-1

Peritoneal macrophages were isolated from WT mice and seeded into 96-well plates. Cells were co-incubated with PBS or eCasp-1 (0.1 μg/mL) in the presence or absence of polymyxin B (15 μg/mL) for 4 h. In another experiment, peritoneal macrophages were isolated from WT mice and seeded into 96-well plates. Cells were stimulated with either PBS or eCasp-1 (0.01, 0.05, 0.1 μg/mL) for 4 h. Peritoneal macrophages were stimulated with PBS or eCasp-1 (0.1 μg/mL) for 4 or 24 h. Furthermore, peritoneal macrophages were isolated from both WT and TLR4^-^/^-^ mice, seeded into 96-well plates, and stimulated with PBS or eCasp-1 (0.1 μg/mL) for 4 h. After stimulation under each condition, culture supernatants were collected and centrifuged at 400 × g for 10 min, and the clarified supernatants were stored at -20 °C for further analysis.

#### C16

Peritoneal macrophages were isolated from WT mice and seeded into 96-well plates. Cells were then treated for 24 h with either PBS, eCasp-1 (0.1 μg/mL), or eCasp-1 pre-incubated for 30 min with C16 (0.1, 1, 10 μg/mL). After treatment, these cell culture supernatants were collected, centrifuged at 400 × g for 10 min, and stored at -20 °C for further analysis.

### *In vivo* experiments

#### Disulfiram

Disulfiram was administered *i.p.* at 50 µg/g BW immediately after gut reperfusion. An equal volume of corn oil was administered *i.p.* as vehicle control. Whole blood and peritoneal cavity fluid were collected 4 h after gut I/R, respectively. Blood samples were centrifuged at 1,000 × g for 15 min to obtain plasma, while peritoneal cavity fluid was centrifuged at 400 × g for 10 min to collect supernatants. All samples were stored at -80 °C for further analysis.

#### eCasp-1

WT mice were *i.p.* injected with eCasp-1 (1, 5 µg/g BW), while control mice received an equal volume of PBS. Blood samples were collected 24 h after injection. In a separate set of experiments, WT mice were administered eCasp-1 at 5 µg/g BW *i.p.*, and blood samples were collected at 4, 24 h post-injection. Additionally, both WT and TLR4^-^/^-^ mice were injected *i.p.* with eCasp-1 (5 µg/g BW), and control animals received PBS. Blood was collected 24 h after treatment. All blood samples were centrifuged at 1,000 × g for 15 min to isolate plasma, which was stored at -80 °C for further analysis.

#### C16

WT mice were subjected to gut I/R received *i.p.* administration of either 10 µg/g BW C16 (treatment) or DMSO in PBS (vehicle), administered immediately after reperfusion. Whole blood and lung tissues were collected at 4 h post-reperfusion for gut I/R. Blood samples were centrifuged at 1,000 × g for 15 min to obtain plasma, which was stored at -80 °C for further analysis. The left lung was collected for wet-to-dry weight ratio or tissue damage evaluation. For the wet-to-dry weight ratio, the lung was used for the analysis immediately after collection. For tissue damage evaluation, the lung was fixed in 10% formalin. The right lung was snap-frozen in liquid nitrogen and stored at -80 °C for further analysis.

### Western blotting

For western blotting, plasma samples were mixed with 4× LDS sample buffer, 10% SDS (Sigma-Aldrich), and PBS in a ratio of 3:5:5:7 (v/v/v/v) and heated at 90 °C for 10 min to denature the proteins. Peritoneal cavity fluid and cell lysate samples were mixed with 4× LDS sample buffer and heated at 99 °C for 5 min. Equal volumes of plasma and peritoneal cavity fluid samples were used for gel loading, and cell lysates samples were adjusted to the same total protein concentration before loading. Proteins were then separated using NuPAGE 4-12% Bis-Tris gels (Invitrogen) and transferred onto PVDF membranes. Immunoblotting was performed with the following primary antibodies according to the manufacturers’ instructions: anti-cleaved caspase-1 (Asp297) (p20 subunit; Cat. No. 4199S, 1:1000) and anti-cleaved caspase-1 (Asp296) (p20 subunit; Cat. No. 89332S, 1:1000) (all from Cell Signaling Technology). Transferrin (Cat. No. 66171-1-Ig, 1:10000, proteinteck, Rosemont, IL) was used as loading controls. After incubation with primary antibodies, membranes were washed three times and subsequently incubated with the corresponding fluorescent secondary antibodies (LI-COR, Lincoln, NE). Bands were visualized using the Odyssey FC Dual-Mode Imaging System 2800 (LI-COR).

### Detection of inflammatory and organ injury markers

Plasma levels of human eCasp-1 were quantified using an ELISA kit (Cat. No. EH5100, FineTest, Wuhan, China) following the manufacturer’s protocol. Plasma, peritoneal cavity fluid, and cell culture supernatant levels of mouse eCasp-1 were quantified using an ELISA kit (Cat. No. AG-45B-0002-KI01, Adipogen, San Diego, CA) following the manufacturer’s protocol. Plasma and cell culture supernatant concentrations of IL-6 and TNFα were measured using ELISA kits (BD Biosciences, Franklin Lakes, NJ). Plasma levels of aspartate aminotransferase (AST), alanine aminotransferase (ALT), and lactate dehydrogenase (LDH) were determined by colorimetric enzymatic assays (Pointe Scientific, Canton, MI). Absorbance was recorded using a Synergy Neo2 microplate reader (Agilent Technologies, Santa Clara, CA) as per the manufacturer’s instructions.

### Computational modeling analysis

Amino acid sequences of eCasp-1 (UniProt ID: P29452) and TLR4 (UniProt ID: Q9QUK6) were retrieved from the UniProt database. In-silico structural modeling was performed using the Iterative Threading ASSEmbly Refinement (I-TASSER) pipeline, which applies a threading-based algorithm to identify optimal structural templates and maximize sequence identity, coverage, and confidence (21). Model refinement was performed through short molecular dynamics simulations (4-fs time step) under mild (0.5 ps) and aggressive (0.8 ps) relaxation protocols to optimize stereochemical quality, including Ramachandran and rotamer statistics. Protein-protein docking between eCasp-1 and TLR4 was conducted using ATTRACT and GRAMMX, which enable flexible side-chain adjustment and global energy landscape mapping to capture both stable and transient interaction states (22, 23). Based on predicted interface residues, a C16 was rationally designed and subjected to flexible docking using pepATTRACT to evaluate potential binding sites and its capacity to disrupt eCasp-1-TLR4 association. Docking interfaces, buried surface areas, and binding energetics were analyzed using PDBePISA, and final complexes were visualized with PyMOL (Schrodinger, Inc., New York, NY) and UCSF Chimera (24, 25). Comparative in-silico docking analyses with or without C16 were performed to validate the predicted inhibitory mechanism and support rational peptide design.

### Surface plasmon resonance analysis

Direct binding between eCasp-1 (p20) and TLR4 was analyzed using surface plasmon resonance (SPR; OpenSPR, Nicoya, Kitchener, ON, Canada). High-sensitivity NTA sensors (HS-NTA; Cat. No. SEN-HS-8-NTA, Nicoya) were employed according to the manufacturer’s WIZARD protocol. All experiments were conducted at 20 °C in running buffer composed of 10 mM HEPES, 150 mM NaCl, and 0.05% P20 (HBS-P20; pH 7.4). Before immobilization, the sensor surface was sequentially cleaned with 10 mM HCl (150 µL) and 350 mM EDTA (150 µL), followed by activation with 40 mM NiCl_2_ (150 µL). eCasp-1 was diluted to 50 µg/mL in 10 mM acetate buffer (pH 5.0) and immobilized onto Channel 2 of the sensor chip. rmTLR4 was injected as an analyte at concentrations of 25, 50, and 100 nM over both Channel 1 (reference) and Channel 2 (ligand) at a flow rate of 30 µL/min. To assess the inhibitory effect of the C16 peptide, eCasp-1 (50 µg/mL) was preincubated with C16 (1 µM) for 30 min at room temperature before immobilization on the sensor surface. rmTLR4 (25–100 nM) was then injected under identical conditions. This design allowed evaluation of whether C16 binding to eCasp-1 interferes with its subsequent interaction with TLR4. Binding reactions were conducted under identical buffer and flow parameters for all conditions. Real-time interaction data were analyzed using TraceDrawer software (Nicoya). Response signals from the reference channel were subtracted from those of the ligand-coated channel to correct for nonspecific binding. Sensorgrams were globally fitted to a 1:1 Langmuir binding model to determine kinetic parameters.

### Reverse transcription-quantitative polymerase chain reaction

Total RNA was isolated from lung tissue using TRIzol reagent (Invitrogen) and reverse-transcribed into cDNA with murine leukemia virus reverse transcriptase (Applied Biosystems, Foster City, CA). Quantitative real-time PCR was performed in a 20 μL reaction containing 5 μM of each forward and reverse primer, 2 μL of cDNA, 7.5 μL of nuclease-free water, and 10 μL of SYBR Green PCR Master Mix (Applied Biosystems). Amplification was conducted on a StepOne-Plus real-time PCR machine (Applied Biosystems) using the following thermal profile: 50 °C for 2 min, 95 °C for 10 min, followed by 40 cycles of 95 °C for 15 sec and 60 °C for 1 min. β-actin was used as an internal control for normalization. Each sample was analyzed in duplicate. Relative mRNA expression was calculated using the ΔΔCT method and expressed as fold change relative to the sham group. Primer sequences are listed in **Supplementary Table 1**.

### Myeloperoxidase assay

Approximately 50–100 mg of lung tissue, pulverized under liquid nitrogen, was homogenized on ice by sonication in potassium phosphate (KPO_4_) buffer containing 0.5% hexadecyltrimethyl-ammonium bromide (Sigma-Aldrich). The homogenates underwent two freeze-thaw cycles and were subsequently centrifuged at 12,000 × g for 15 min at 4 °C to collect the supernatant. MPO activity was assessed in a 96-well plate by incubating samples with phosphate buffer containing o-dianisidine hydrochloride (Sigma-Aldrich) and hydrogen peroxide (Thermo Fisher Scientific). Absorbance was measured at 460 nm every minute over a 5 min period using a Synergy Neo2 plate reader (Agilent Technologies). MPO activity was defined as the change in absorbance per minute and expressed as units per gram of tissue.

### Lung wet and dry weight

Lung wet and dry weights were measured as previously described (26). Briefly, the left lung was excised and immediately weighed to determine the wet lung weight. The lung samples were then dried at 65 °C for 48 h until a constant weight was achieved, after which they were weighed again to determine the dry weight. The wet-to-dry weight was calculated as an indicator of pulmonary edema.

### Lung histology and TUNEL

At the time of tissue collection, left lungs were fixed in 10% neutral-buffered. Fixed tissues were subsequently embedded in paraffin, sectioned at 4 µm thickness, and stained with hematoxylin and eosin (H&E). Lung injury score was evaluated by light microscopy and scored using the American Thoracic Society criteria (27). Briefly, sections were assessed blindly across 10 randomly selected fields at ×200 magnification for the presence of proteinaceous debris in airspaces, septal thickening, and neutrophil infiltration in alveolar and interstitial spaces, with each parameter scored from 0 to 1 and averaged for analysis. For the detection of apoptotic cells, terminal deoxynucleotidyl transferase dUTP nick end labeling (TUNEL) staining was performed on 4 µm lung sections using a fluorescein *in situ* cell death detection kit (Roche Diagnostics, Indianapolis, IN) according to the manufacturer’s protocol. Nuclei were counterstained with 4′,6-diamidino-2-phenylindole (DAPI) (Vectashield Antifade Mounting Media; H-2000, Vector Laboratories). TUNEL-positive cells were quantified in 10 randomly selected fields per section under a fluorescence microscope at ×200 magnification, using ImageJ (version 2.1.0/51; Fiji software).

### Survival study

Mice underwent gut I/R as described above and received a single *i.p.* dose of C16 (10 µg/g BW) or an equivalent volume of vehicle. Mice were monitored every 4–8 h for 40-h. An event was defined as either spontaneous death or fulfillment of at least two humane endpoint criteria, including minimal to absent movement, abnormal respiration, a grimace score of 2, a body condition score below 3, weight loss exceeding 20%, or the presence of bloody diarrhea.

### Statistical analysis

Data are presented as mean ± standard error of the mean (SEM). Statistical comparisons between two groups, unpaired two-tailed Student’s t-test was employed. For comparisons among multiple groups were performed using one-way analysis of variance (ANOVA) followed by the Student-Newman-Keuls (SNK) *post hoc* test. Survival analyses were conducted using the Kaplan-Meier method, and differences between groups were assessed by the log-rank test. A p-value of < 0.05 was considered statistically significant. All statistical analyses were performed using GraphPad Prism software (version 10.4.1; GraphPad Software, San Diego, CA).

## Results

### eCasp-1 is elevated in critically ill patients and animals with gut I/R

**Figure 1:**
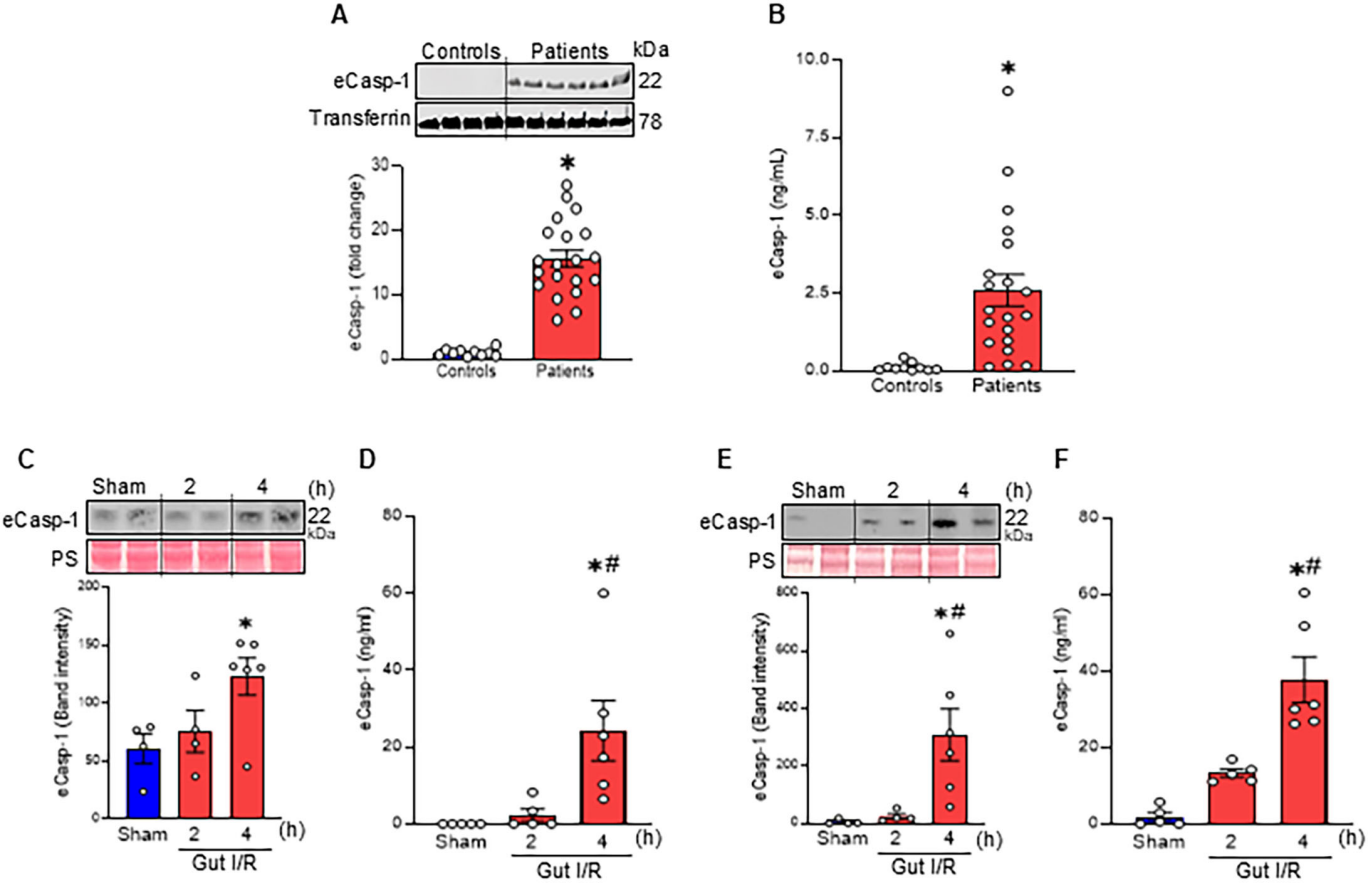
eCasp-1 is elevated in patients and murine gut I/R. **(A, B)** Plasma eCasp-1 levels in healthy control and SICU patients were analyzed by **(A)**Western blot and **(B)** ELISA. Experiments were repeated at least 4 times, and data represent the combined results. Data are expressed as mean ± SEM (n = 10–20 samples/group) and compared by a student’s t-test (^*^p < 0.05 vs. Controls). **(C-F)** WT mice were subjected to 60 min of gut I/R, and **(C, D)** plasma and **(E, F)** peritoneal cavity fluid were collected at 2 or 4 h after reperfusion. eCasp-1 levels were measured by Western blot and ELISA. Experiments were performed 3 times, and all data were used for analysis. Data are expressed as mean ± SEM (n = 6–9 samples/group) and compared by one-way analysis of variance and Student-Newman-Keuls method [^*^p < 0.05 vs. Sham; ^#^p < 0.05 vs. Gut I/R (2 h)].

During inflammation or hypoxia, different sensor proteins are recruited to activate the inflammasome pathway and cleave procaspase-1, generating a p10/p20 heterodimer (2, 28, 29). This heterodimer then further dimerizes to form active (tetrameric) intracellular Casp-1. Interestingly, using Western blot analysis, we detected high levels of the p20 subunit of Casp-1 in the plasma of de-identified SICU patients at North Shore University Hospital, whereas it was undetectable in the blood of healthy subjects (**Figure 1A**). Active Casp-1 is commonly assessed via detection of the p20 subunit by Western blotting (19, 30). Therefore, in this study, we specifically refer to the p20 subunit as extracellular Casp-1 (eCasp-1). We further confirmed the presence of eCasp-1 using ELISA, which revealed significantly higher levels in the plasma of SICU patients (**Figure 1B**). These findings demonstrate the discovery of eCasp-1 in the blood of critically ill patients. In mice subjected to gut I/R, eCasp-1 levels were markedly elevated in both plasma and peritoneal cavity fluid compared with sham controls in a time-dependent manner. In plasma, eCasp-1 increased from 1.2- to 2-fold by Western blot and from 2.3- to 24.2-fold by ELISA between 2 h and 4 h post-reperfusion. In peritoneal cavity fluid eCasp-1 was more significantly elevated from 9.9- and 21.2-fold by Western blot and 7- and 19.9-fold by ELISA over the same period (**Figures 1C–F**).

### eCasp-1 is released via GSDMD-dependent processes

**Figure 2:**
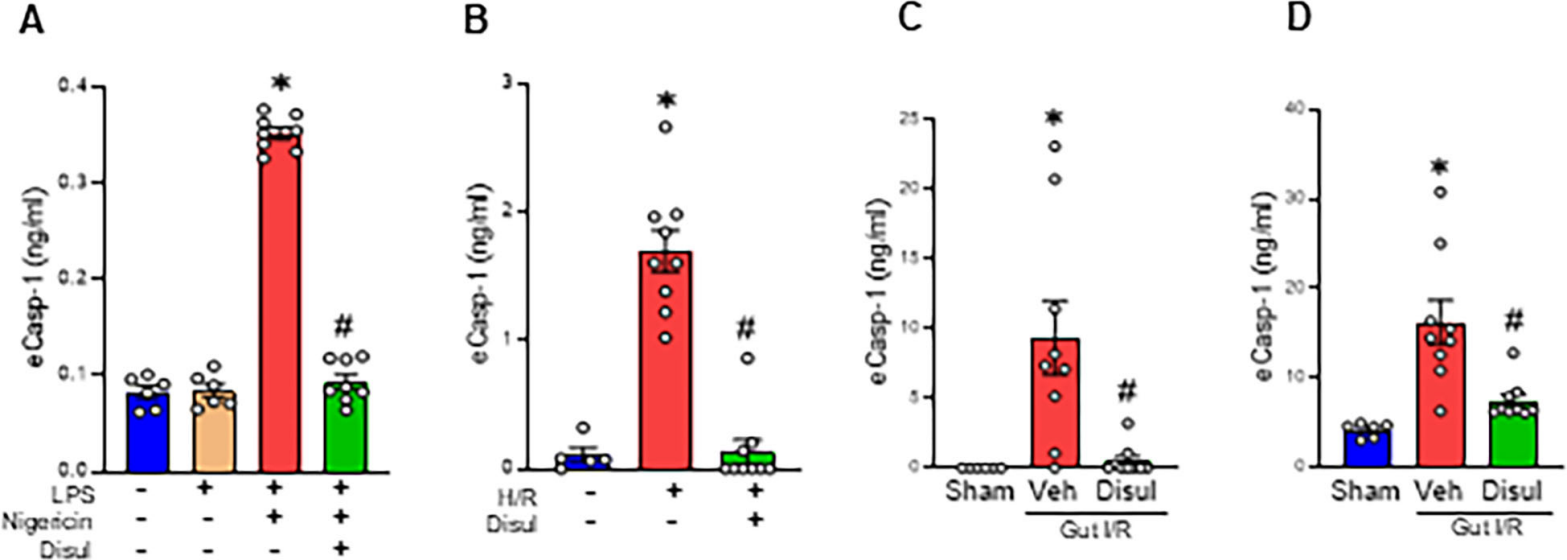
eCasp-1 release involves GSDMD-dependent membrane processes **(A, B)** WT peritoneal macrophages were pretreated with or without Disul (5 µM) and **(A)** stimulated with LPS followed by nigericin or **(B)** exposed to H/R. eCasp-1 levels in the culture supernatants were measured by ELISA. **(C, D)** Gut I/R was induced in WT mice for 60 min, and Veh or Disul (50 µg/g BW) was injected *i.p.* at reperfusion. **(C)** Plasma and **(D)** peritoneal cavity fluid were collected 4 h after reperfusion and eCasp-1 measured by ELISA. Experiments were performed 2–3 times, and all data were used for analysis. Data are expressed as mean ± SEM (n = 6–9 samples/group) and compared by one-way analysis of variance and Student-Newman-Keuls method [^*^p < 0.05 vs. PBS, Sham; ^#^p < 0.05 vs. (+)LPS (+)nigericin (-)Disul, (+)H/R (-)Disul, Veh]. Disul, Disulfiram; Veh, Vehicle.

Given that GSDMD forms membrane pores that could lead lytic or non-lytic cellular processes during inflammasome activation, we hypothesized that eCasp-1 release is associated with GSDMD activation (2). We examined the effects of the GSDMD inhibitor disulfiram on eCasp-1 release. In peritoneal macrophages primed with LPS, subsequent stimulation with nigericin to activate the NLRP3 inflammasome resulted in a 4.3-fold increase in eCasp-1 release compared with PBS-treated controls, whereas disulfiram treatment markedly suppressed this increase by 73.4% (**Figure 2A**). Under hypoxia/reoxygenation (H/R) conditions mimicking gut I/R stress with peritoneal macrophages, eCasp-1 levels were significantly elevated by 15.9-fold compared to the control group but were reduced by 92.1% following disulfiram treatment (**Figure 2B**). We next turned to intestinal organoids as an *in vitro* model to study the release of eCasp-1 from intestinal epithelial cells during gut I/R injury. H/R condition organoids showed a 6.3-fold increase in eCasp-1 compared with untreated normoxic cells, whereas disulfiram treatment reduced this increase by 80.7% (**Supplementary Figure 1**). In the gut I/R model, plasma and peritoneal cavity eCasp-1 levels increased 9.3- and 3.9-fold, respectively, at 4 h after reperfusion, but were significantly reduced by 94.6% and 54.3%, respectively, following disulfiram administration (**Figures 2C, D**).

### eCasp-1 is a new DAMP and exacerbates inflammation and tissue injury

**Figure 3:**
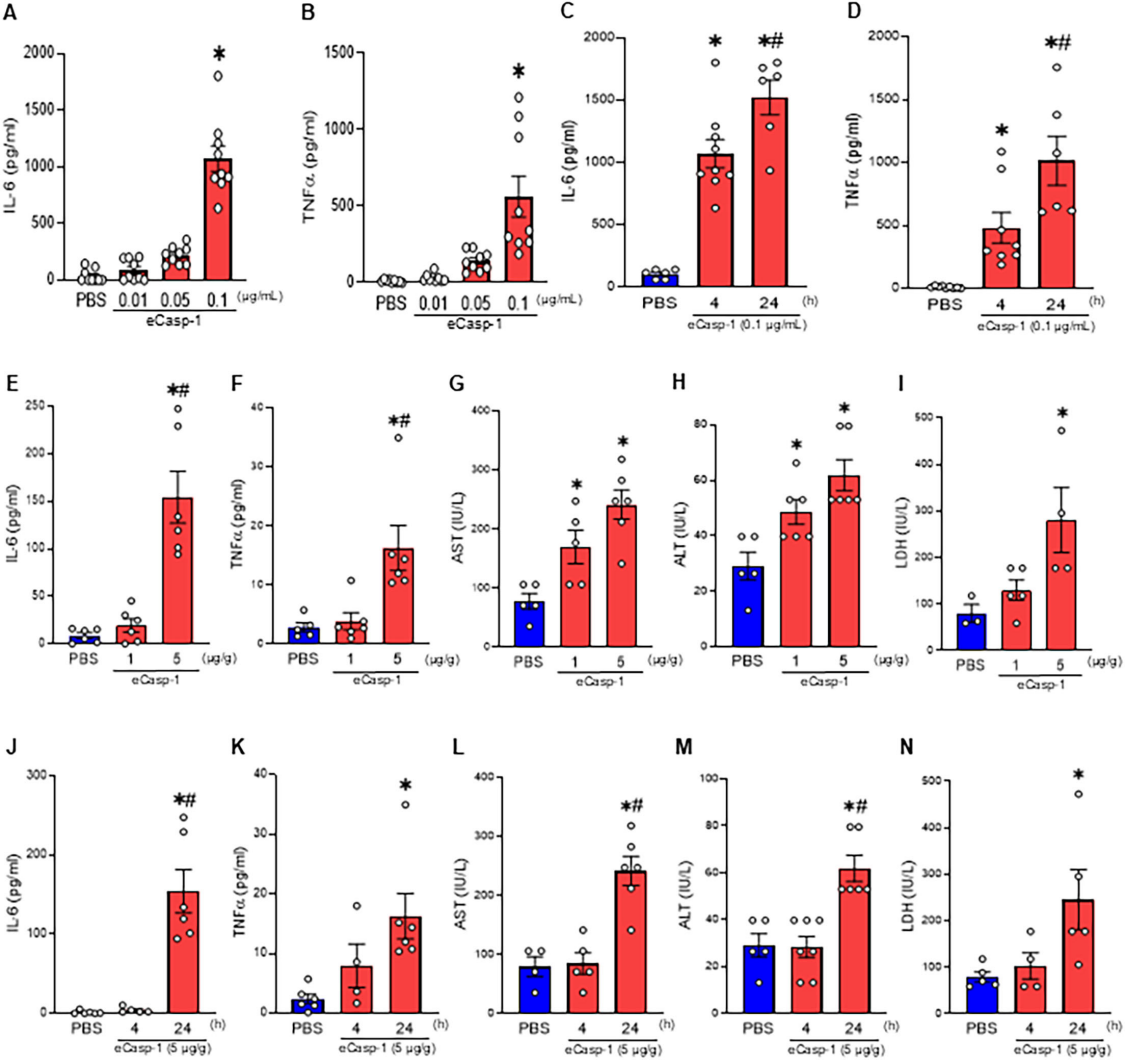
eCasp-1 functions as a DAMP to induce cytokine release and organ injury. **(A, B)** WT peritoneal macrophages were stimulated with increasing concentrations of eCasp-1 (0.01, 0.05, 0.1 µg/ml) for 4 h, and **(A)** IL-6 and **(B)** TNFα levels in the culture supernatants were measured by ELISA. **(C, D)** peritoneal macrophages were stimulated with eCasp-1 (0.1 µg/ml) for 4 or 24 h, and **(C)** IL-6 and **(D)** TNFα were quantified by ELISA. **(E-I)** WT mice received *i.p.* injections of eCasp-1 (1, 5 µg/g BW). Plasma was collected at 24 h to measure **(E)** IL-6, **(F)** TNFα, **(G)** AST, **(H)** ALT, and **(I)** LDH as indicators of systemic inflammation and organ injury. **(J-N)** WT mice were injected with eCasp-1 (5 µg/g BW), and plasma was collected at 4 h or 24 h to assess temporal changes in **(J)** IL-6, **(K)** TNFα, **(L)** AST, **(M)** ALT, and **(N)** LDH. Experiments were performed 3 times, and all data were used for analysis. Data were expressed as mean ± SEM (n = 3–9 samples/group) and compared by one-way analysis of variance and Student-Newman-Keuls method [^*^p < 0.05 vs. PBS; ^#^p < 0.05 vs. eCasp-1 (4 h), eCasp-1 (1 μg/g)].

We examined the effects of exogenous eCasp-1 in both *in vitro* and *in vivo* settings. When peritoneal macrophages were stimulated with eCasp-1, the concentrations of IL-6 and TNFα in the culture supernatants significantly increased in both dose- and time-dependent manner (**Figures 3A–D**). Polymyxin B, which binds to the lipid A portion of LPS to neutralize its effects, did not suppress eCasp-1 induced cytokine production, confirming that the responses were indeed attributable to eCasp-1 itself rather than LPS contamination (**Supplementary Figures 2A, B**). Consistent with these findings, *i.p.* administration of eCasp-1 to normal mice caused a dose-dependent increase in plasma IL-6 and TNFα levels, concomitant with higher levels of AST, ALT, and LDH compared with PBS-injected mice (**Figures 3E–I**). Furthermore, time-course analysis further revealed that these inflammatory and injury markers progressively increased following eCasp-1 administration, demonstrating a sustained proinflammatory and tissue-damaging effect (**Figures 3J–N**). These findings demonstrate that eCasp-1 acts as a new DAMP, functioning as a potent extracellular mediator that promotes inflammation and organ injury.

### eCasp-1 strongly interacts with TLR4, exacerbating inflammation

**Figure 4:**
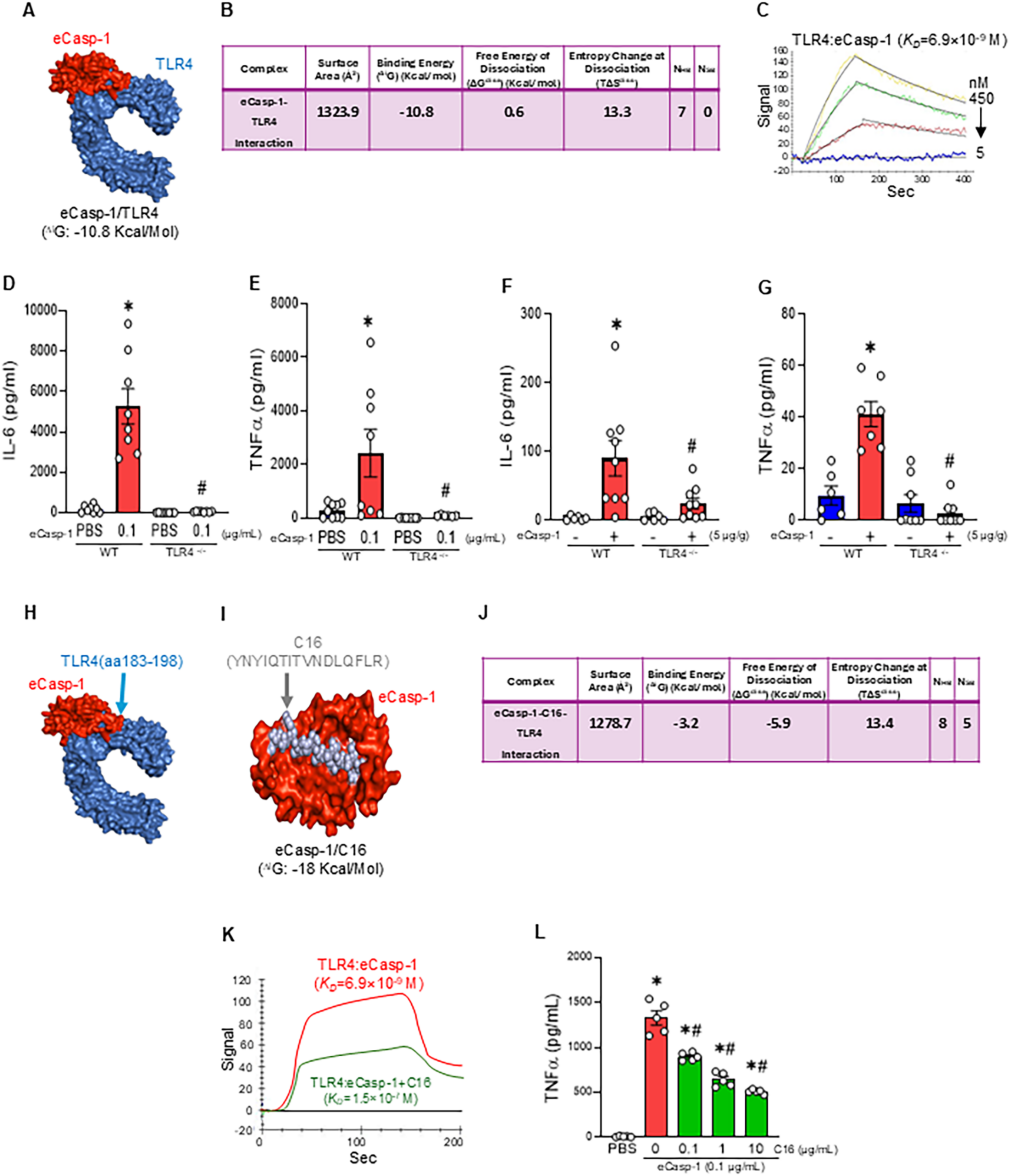
eCasp-1 binds to TLR4 to drive inflammation, which is effectively suppressed by the novel peptide C16. **(A, B)** Computational modeling predicted a strong interaction between eCasp-1 (red) and TLR4 (blue). **(C)** SPR analysis on the binding of eCasp-1 to TLR4 *in vitro*. **(D, E)** WT and TLR4^-/-^ peritoneal macrophages were treated with PBS or eCasp-1 (0.1 µg/ml) for 4 h. **(D)** IL-6 and **(E)** TNFα levels in the supernatants were measured by ELISA. **(F, G)** WT and TLR4^-/-^ mice received *i.p.* injections of PBS or eCasp-1 (5 µg/g BW), and plasma was collected 24 h later to measure **(F)** IL-6 and **(G)** TNFα. **(H)** In-silico analysis identified a putative binding site for mouse eCasp-1 (red) on the extracellular domain of TLR4 (blue). **(I)** C16 (silver), a 16-amino-acid peptide mimic, was designed based on the predicted binding interface and exhibited strong binding to eCasp-1 (red). **(J)** Computational modeling predicted a potential interaction between the eCasp-1-C16 complex and TLR4. **(K)** SPR analysis of eCasp-1 binding to TLR4 in the presence or absence of C16. **(L)** WT peritoneal macrophages were treated with eCasp-1 (0.1 µg/mL) with increasing concentrations of C16 (0.1, 1, 10 µg/mL) for 4 h, and TNFα levels in the supernatants were measured by ELISA. Experiments were repeated 2–3 times and all the data obtained were used for analysis. Data were expressed as mean ± SEM (n = 5–9 samples/group) and compared by one-way analysis of variance and Student-Newman-Keuls method (^*^p < 0.05 vs. WT PBS; ^#^p < 0.05 vs. WT with eCasp-1, (+)eCasp-1 (-)C16).

We next aimed to investigate whether eCasp-1 interacts with TLR4, a key receptor, promoting innate immune response. Computational modeling predicted a strong binding interaction between eCasp-1 and TLR4, with a calculated binding free energy of ΔiG = -10.8 kcal/mol (**Figures 4A, B**). Consistent with this finding, SPR analysis revealed a strong binding affinity between eCasp-1 and TLR4, exhibiting a dissociation constant (*K_D_*) of 6.9×10^–9^ M (**Figure 4C**). To determine whether this interaction contributed to eCasp-1-induced inflammation, we stimulated peritoneal macrophages derived from WT and TLR4^-^/^-^ mice with eCasp-1. Interestingly, we revealed that eCasp-1 markedly increased IL-6 and TNFα secretion in WT macrophages, whereas cytokine release was nearly at the basal level in TLR4^-^/^-^ cells (**Figures 4D, E**). *In vivo* administration of eCasp-1 induced robust systemic inflammation, as plasma IL-6 and TNFα levels were significantly elevated by approximately 29- and 4.3-fold, respectively, in WT mice, while these increases were significantly attenuated by 72.4% and 93%, respectively in TLR4^-^/^-^ mice (**Figures 4F, G**). These findings demonstrate that eCasp-1 directly binds to TLR4 on immune cells and triggers cytokine release, acting as a DAMP to promote inflammation.

### C16, a novel peptide, by targeting eCasp-1/TLR4, attenuates inflammation in macrophages

We then aimed to develop a peptide inhibitor to disrupt eCasp-1-TLR4 interaction. Using an in-silico approach, we identified a putative binding site for mouse eCasp-1 on the mouse extracellular domain of TLR4 (aa183-198) (**Figure 4H**), which exhibits significant sequence homology with the extracellular domain of human TLR4. Based on this information, we designed a 16-aa peptide mimic, C16 (YNYIQTITVNDLQFLR), and confirmed its strong binding to eCasp-1 (ΔiG = -18 kcal/mol) (**Figure 4I**). Furthermore, in-silico modeling predicted a potential interaction between the eCasp-1-C16 complex and TLR4, with a calculated binding free energy of ΔiG = -3.2 kcal/mol (**Figure 4J**). We then studied whether the presence of C16 can inhibit the binding of eCasp-1 to TLR4. In SPR assays, the presence of C16 dramatically reduced the binding affinity between eCasp-1 and TLR4, with the *K_D_* increasing from 6.9x10^–9^ to 1.5x10^–7^ M, which corresponds to an approximately 218-fold decrease in affinity (**Figure 4K**). Consistently, cell-based assays using peritoneal macrophage demonstrated that C16 significantly and dose-dependently suppressed eCasp-1-induced TNFα release from macrophages (**Figure 4L**). These results identify C16 as a novel eCasp-1 antagonist peptide that effectively interferes with eCasp-1’s TLR4 interaction and suppresses cytokine production by macrophages.

### C16 attenuates systemic inflammation and ALI and improves survival in gut I/R

**Figure 5:**
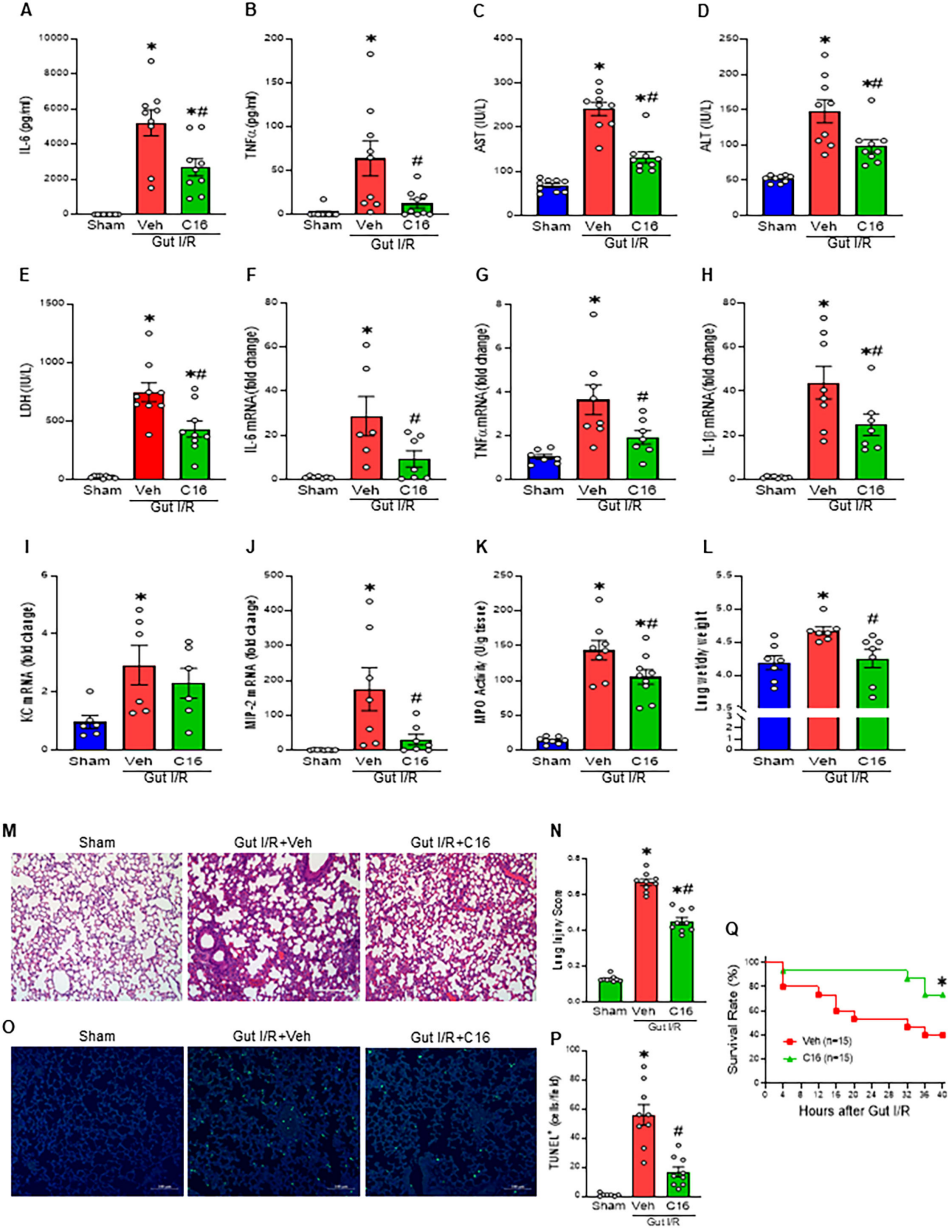
Treatment with C16 attenuates systemic inflammation, lung injury, and mortality in gut I/R. **(A-E)** Plasma was collected 4 h after reperfusion, and **(A)** IL-6, **(B)** TNFα, **(C)** AST, **(D)** ALT, and **(E)** LDH levels were quantified by ELISA. **(F-K)** Right lung tissues were harvested 4 h after reperfusion for analysis of **(F)** IL-6, **(G)** TNFα, **(H)** IL-1β, **(I)** KC, and **(J)** MIP-2 mRNA expression, and **(K)** MPO activity. **(L)** The wet-to-dry weight ratio of the left lung was determined as an indicator of pulmonary edema. **(M)** Representative H&E-stained lung sections (magnification ×200; scale bar. 200 μm). **(N)** Lung injury was scored by a blinded investigator using a standardized histopathological scoring system. **(O)** Representative TUNEL staining images showing apoptotic (green) and nuclear (blue) signals in lung sections (magnification ×200; scale bar. 100 μm). **(P)** Quantification of TUNEL-positive cells in lung sections. **(Q)** Kaplan-Meier survival curves showing cumulative survival over 40-hour after gut I/R in Veh-injected and C16-treated groups. Experiments were performed 3 times, and all data were used for analysis. Data were expressed as mean ± SEM (n = 6–9 samples/group) and compared by one-way analysis of variance and Student-Newman-Keuls method (^*^p < 0.05 vs. Sham; ^#^p < 0.05 vs. Veh). Survival rates were analyzed by the Kaplan-Meier estimator using a log-rank test (n = 15 mice/group, ^*^p < 0.05 vs. Veh). Veh, Vehicle.

**Figure 6:**
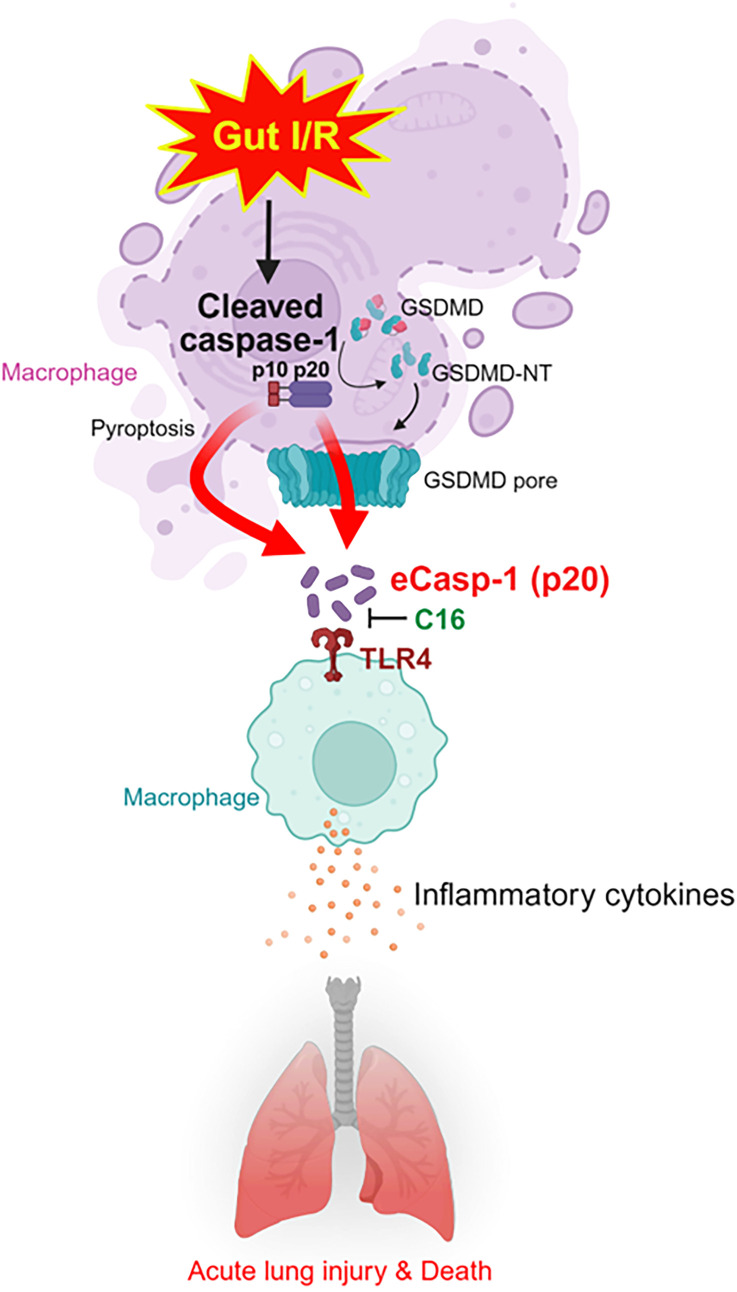
Summary of findings. In gut I/R injury, inflammasomes activation promotes the cleavage of caspase-1 and the extracellular release of its p20 subunit through GSDMD-dependent membrane processes. This release may occur in association with GSDMD pore formation as well as membrane disruption during lytic cell death (pyroptosis). Once released, extracellular caspase-1 (eCasp-1) acts as a potent DAMP by binding to TLR4, thereby amplifying release of inflammatory cytokines, aggravating lung injury. Therapeutic intervention with the inhibitory peptide C16, which specifically blocks the eCasp-1-TLR4 interaction, effectively attenuates systemic inflammation and improves survival outcomes. I/R, Ischemia-reperfusion; GSDMD, Gasdermin-D; eCasp-1, Extracellular caspase-1; DAMP, Damage-associated molecular pattern; TLR4, Toll-like receptor 4.

In the gut I/R model, mice received an *i.p.* injection of vehicle or C16 immediately upon reperfusion. Systemic inflammation was evaluated by measuring plasma cytokines and organ injury markers. In vehicle-injected mice, plasma IL-6 and TNFα levels increased 3.5-fold and 2.8-fold compared with sham controls, accompanied by significant elevations in AST, ALT, and LDH levels, which rose 32.5-, 45.3-, and 33.2-fold, respectively. Administration of C16 significantly attenuated these changes, reducing IL-6, TNFα, AST, ALT, and LDH levels by 48.6%, 80.8%, 45.3%, 33.2%, and 42.3%, respectively (**Figures 5A–E**). We next examined the induction of eCasp-1-mediated inflammation in the lung, a key remote organ target in gut I/R. In vehicle-injected mice, pulmonary IL-6, TNFα, and IL-1β mRNA levels significantly increased 26.6-, 3.5-, and 40.9-fold, respectively, relative to sham controls. C16 treatment markedly reduced these increases, lowering IL-6, TNFα, and IL-1β expression by 67.4%, 47.0%, and 43.4%, respectively (**Figures 5F–H**). We also evaluated keratinocyte chemoattractant (KC), macrophage inflammatory protein-2 (MIP-2), and myeloperoxidase (MPO) activity in the lung. In vehicle-injected mice, KC and MIP-2 levels increased 3- and 152.5-fold, respectively, and MPO activity increased 10.1-fold. C16 treatment significantly reduced these values by 21.2%, 82.3%, and 26.7%, respectively (**Figures 5I–K**). Pulmonary edema, assessed by wet-to-dry lung weight ratio, was also markedly attenuated by C16 treatment following gut I/R (**Figure 5L**). Histopathological evaluation using H&E staining and the American Thoracic Society scoring system, which considers alveolar congestion, protein deposition, neutrophil infiltration, hemorrhage, and epithelial damage, revealed severe alveolar disruption and inflammation in vehicle-injected mice (27). In contrast, C16-treated mice exhibited preservation of alveolar structure and a 32.6% reduction in lung injury scores (**Figures 5M, N**). TUNEL assays demonstrated that apoptotic cell numbers, which increased 55.9-fold in vehicle-injected mice compared to sham, were reduced by 70% with C16 treatment in gut I/R mice (**Figures 5O, P**). To assess the potential therapeutic impact of C16 on survival, we monitored mortality in the gut I/R model. Vehicle-injected mice exhibited a 40-h survival rate of 40%, whereas C16 administration significantly improved survival to 80% (**Figure 5Q**). Collectively, these findings indicate that C16 markedly reduces systemic and pulmonary inflammation, limits tissue damage, and enhances survival in gut I/R (**Figure 6**).

## Discussion

In this study, we discovered eCasp-1 as a new DAMP that exacerbates inflammation and ALI, deadly conditions frequently caused by gut I/R. We first established the clinical relevance of eCasp-1 by demonstrating markedly elevated plasma levels in SICU patients compared with healthy controls. Critically ill deidentified patients admitted to the SICU exhibit heterogeneous etiologies including ischemia, infection, trauma, and post-resuscitation injury encompassing both sterile and non-sterile inflammation (31, 32). To reflect this clinical spectrum, we utilized complementary murine models of gut I/R, characterized by ischemia-induced sterility and post-reperfusion infection due to bacterial translocation (9–11). This experimental strategy enabled us to disease-specific mechanisms of eCasp-1 release and its systemic impact. This models ultimately lead to ALI, a hallmark of multi-organ failure in critical illness, underscoring the translational relevance of our findings (12). Using this models, we found that eCasp-1 is secreted dependent on the GSDMD pore-forming process and functions as a new DAMP, driving systemic inflammation, ALI, and mortality. While this observation aligns with previous reports describing increased circulating DAMPs, such as eCIRP and HMGB1, during gut I/R, it distinctly reveals that an intracellular protease long recognized solely for its catalytic role within the inflammasome can exert a proinflammatory function in the extracellular milieu (33, 34).

Given that eCasp-1 release appears to involve GSDMD-dependent membrane processes, we next considered the upstream mechanisms regulating GSDMD activation. GSDMD cleavage can be mediated by both caspase-1 and caspase-11 through canonical and non-canonical inflammasome pathways; however, the specific upstream caspase responsible in this setting was not determined in the present study (1, 35). Thus, while our data supports a role for GSDMD-dependent membrane processes in eCasp-1 release during ischemic stress, the relative contribution of individual inflammasome pathways remains to be clarified. Collectively, these findings extend the current understanding of inflammasome-associated responses by identifying GSDMD-mediated membrane processes as a potential contributor to eCasp-1 release, with implications for systemic inflammation, organ injury, and host survival.

Given this mechanistic context, it was critical to verify the molecular identity of the extracellular caspase detected in our experiments. Because caspase family members share structural similarities and may be activated under inflammatory conditions, distinguishing between caspase-1 and caspase-11 is particularly important (1, 35, 36). In the present study, eCasp-1 was measured using approaches designed to specifically recognize the cleaved caspase-1 p20 fragment. Western blot analysis employed an antibody directed against the Asp297 (human) and Asp296 (mouse) cleavage site, selectively detecting the caspase-1 p20 subunit, while ELISA quantification was performed using monoclonal antibodies specific for caspase-1 p20. These complementary approaches support the interpretation that the extracellular caspase detected represents activated caspase-1. However, because the activation status and extracellular release of caspase-11 were not directly assessed, its potential contribution to the detected extracellular signals cannot be completely excluded. Future studies using caspase-1-deficient models or caspase-11-specific validation approaches will be necessary to clarify the relative contributions of these inflammatory caspases.

Building on this molecular characterization, we next considered the structural form of caspase-1 and its implications for extracellular release. The structural form of caspase-1 is central to understanding its immunological function. GSDMD pores, estimated to be 10–20 nm in diameter, theoretically permit the passage of molecules of approximately 17–20 kDa (5, 8, 37). Cleaved caspase-1 consists of a 20-kDa large subunit (p20) and a 10-kDa small subunit (p10), which together form an active tetramer (two p20/p10 dimers) of about 60-kDa (38). Given these size and steric constraints, the intact caspase-1 tetramer is unlikely to pass through GSDMD pores. Previous studies have shown that active caspase-1 rapidly loses enzymatic activity after substrate cleavage, indicating conformational lability of the complex (39, 40). We therefore propose that inactivation is accompanied by structural rearrangement or partial dissociation, generating smaller species, such as p20/p10 heterodimers or p20 monomers which could potentially move through GSDMD pores. However, it is important to emphasize that our experiments were not specifically designed to determine whether eCasp-1 release represents a regulated secretion process directly through GSDMD pores, or occurs secondarily as a consequence of membrane disruption and lytic cell death, such as pyroptosis. Indeed, GSDMD pore formation is closely linked to cell membrane permeabilization, and eCasp-1 may be released in conjunction with this process rather than by selective transport (2, 5). In our study, pharmacological inhibition of GSDMD with disulfiram markedly reduced eCasp-1 levels in both *in vitro* and *in vivo* models, supporting a role for GSDMD-dependent pore membrane processes in eCasp-1 release. Taken together, these findings suggest that GSDMD-mediated membrane perturbation represents one contributing pathway for eCasp-1 release, while other mechanisms, including lytic cell death or additional unknown processes, may also contribute. This interpretation aligns with the concept that eCasp-1 release is likely multifactorial and context-dependent, rather than solely dictated by direct passage through GSDMD pores.

Our study further indicates that eCasp-1 binds to TLR4 with high affinity and triggers downstream cytokine release and inflammatory amplification. In our experiments, peritoneal macrophages were stimulated with eCasp-1 with or without polymyxin B, confirming that cytokine release was independent of endotoxin contamination and attributable to eCasp-1 itself. To mechanistically validate TLR4 as the primary receptor for eCasp-1, we employed a multi-tiered approach. First, we employed in-silico modeling to examine the interaction between eCasp-1 and TLR4, given that most DAMPs signal through TLR4 and that TLR4 functions as a heterogeneous ligand-binding receptor (41). Second, this interaction was quantitatively validated using SPR, revealing a strong binding affinity (*K_D_* = 6.9 × 10^-9^ M) reminiscent of antigen-antibody interactions (42, 43). Third, using peritoneal macrophages from TLR4^-/-^ mice, we demonstrated that the cytokine inducing effect of eCasp-1 was completely abrogated, confirming that TLR4 is the pivotal receptor mediating eCasp-1 signaling in these cells and under *in vivo* conditions. Nevertheless, the contribution of other immune and non-immune cell types, which may express low or no TLR4, warrants further investigation, as eCasp-1 could engage additional receptors depending on the cellular context.

TLR4 signaling requires formation of a receptor complex with its co-receptor MD-2, in which the hydrophobic pocket of MD-2 accommodates the lipid chains of LPS and promotes receptor dimerization (44). Therefore, understanding the structural basis of the eCasp-1-TLR4 interaction is critical for interpreting how this DAMP initiates inflammatory signaling. In the present study, computational docking analysis predicted that eCasp-1 interacts with the extracellular domain of TLR4 rather than with the MD-2 lipid-binding pocket. This interaction was experimentally supported by SPR, which demonstrated direct high-affinity binding between eCasp-1 and TLR4. Because caspase-1 is a soluble protease lacking lipid moieties or hydrophobic acyl chains, it is structurally unlikely to occupy the MD-2 lipid-binding cavity used by LPS (45). These observations suggest that eCasp-1 most likely engages TLR4 through a protein-protein interaction interface on the receptor ectodomain that is distinct from the canonical LPS-binding site. Several endogenous DAMPs have been reported to activate TLR4 through similar protein-based interactions. For example, the alarmin HMGB1 can interact with the TLR4/MD-2 complex and induce inflammatory signaling through receptor interfaces distinct from the MD-2 lipid-binding pocket (46). However, the precise binding interface of eCasp-1 within the fully assembled TLR4-MD-2 dimer has not yet been structurally resolved. In the current study, we primarily investigated whether eCasp-1 binds to TLR4 and confirmed that TLR4 deficiency inhibits its effects in cellular and *in vivo* models. Therefore, understanding whether eCasp-1 binding directly influences TLR4 dimerization or alters ligand accessibility to MD-2 will be a critical focus for future studies. Based on these findings, we next investigated whether selective blockade of the p20-TLR4 interaction could attenuate eCasp-1-driven systemic inflammation and mortality.

Given the high-affinity interaction between eCasp-1 and TLR4, we sought to delineate the amino acid sequence within TLR4 responsible for this binding and to develop a targeted inhibitory strategy. Using a decoy-based approach, we designed a 16-amino-acid peptide (C16) that mimics this critical TLR4 interface. C16 effectively disrupted the eCasp-1-TLR4 interaction and significantly attenuated eCasp-1-induced inflammatory responses in macrophages, highlighting the pathogenic importance of this signaling axis. Unlike global TLR4 inhibition, which may impair host defense and has not improved outcomes in sepsis clinical trials, this ligand-targeted strategy offers a more selective approach to modulating DAMP-driven inflammation (47, 48).

Because TLR4 is classically activated by LPS, it was important to assess whether the observed anti-inflammatory effects of C16 could be attributed to direct LPS neutralization. Sequence analysis of C16 (YNYIQTITVNDLQFLR) reveals a predominantly hydrophilic composition with minimal cationic charge, lacking the amphipathic structure and clustered positively charged residues typically required for interaction with the negatively charged lipid A moiety of LPS (49). These physicochemical features argue against a canonical LPS-neutralizing mechanism. Consistent with this interpretation, computational docking and surface plasmon resonance analyses support a model in which C16 binds eCasp-1 and interferes with its interaction with TLR4. However, because the effects of C16 were not directly evaluated in LPS-driven models, further studies will be necessary to fully establish its specificity in the context of endotoxin-mediated inflammation.

Our study may exhibit some limitations. First, several aspects of eCasp-1 remain incompletely characterized, including its structural heterogeneity, the presence and specific role of the p10 subunit, its enzymatic activity, and potential alternative release mechanisms such as membrane rupture or exosome-like vesicles. Second, although we used cleavage site-specific monoclonal antibodies and ELISA systems designed to selectively detect caspase-1 p20, the structural similarity between caspase-1 and caspase-11 p20 fragments raises the possibility of cross-reactivity. Therefore, the contribution of caspase-11 to the detected extracellular signals cannot be completely excluded. Third, pharmacological optimization of C16, including its blood half-life, tissue distribution, and target engagement, is required. Future studies are essential to determine whether modulation of the eCasp-1-TLR4 axis can selectively attenuate pathological inflammation without impairing host defense, and to explore whether other protease fragments share similar extracellular signaling capabilities.

In conclusion, this study identifies cleaved caspase-1 (p20) as a new DAMP released in association with GSDMD-dependent pore membrane processes, triggering potent inflammatory responses via TLR4. The C16 peptide selectively inhibits eCasp-1, mitigating systemic inflammation, acute lung injury, and mortality. Collectively, these findings reinforce eCasp-1 as a critical extracellular mediator linking inflammasome activity and DAMP signaling in acute inflammation.

## Data Availability

The original contributions presented in the study are included in the article/**Supplementary Material**. Further inquiries can be directed to the corresponding authors.

## Glossary

ALI: acute lung injury
ALT: alanine aminotransferase
ANOVA: analysis of variance
AST: aspartate aminotransferase
ATP: adenosine triphosphate
BW: body weight
cDNA: complementary DNA
CO₂: carbon dioxide
C16: compound 16
DAMP: damage-associated molecular pattern
DAPI: 4′,6-diamidino-2-phenylindole
Disul: disulfiram
DMSO: dimethyl sulfoxide
dUTP: deoxyuridine triphosphate
eCasp-1: extracellular caspase-1
EDTA: ethylenediaminetetraacetic acid
ELISA: FBS, fetal bovine serum
FDA: U.S. Food and Drug Administration
GSDMD: gasdermin D
GSDMD-NT: N-terminal fragment of GSDMD
H&E: hematoxylin and eosin
HEPES: 4-(2-hydroxyethyl)-1-piperazineethanesulfonic acid
HMGB1: high mobility group box 1
H/R: hypoxia/reoxygenation
ID: identification
IL: interleukin
I/R: ischemia-reperfusion
IRB: institutional review board
KC: keratinocyte-derived chemokine
KPO₄: potassium phosphate
LDH: lactate dehydrogenase
LDS: lithium dodecyl sulfate
LPS: lipopolysaccharide
MIP-2: macrophage inflammatory protein-2
MPO: myeloperoxidase
mRNA: messenger RNA
N₂: nitrogen
NIH: National Institutes of Health
NLRP3: NOD-, LRR- and pyrin domain-containing protein 3
O₂: oxygen
PBS: phosphate-buffered saline
PCR: polymerase chain reaction
PVDF: polyvinylidene fluoride
RIPA: radioimmunoprecipitation assay
rm: recombinant mouse
SDS: sodium dodecyl sulfate
SEM: standard error of the mean
SICU: surgical intensive care unit
SIRS: systemic inflammatory response syndrome
SMA: superior mesenteric artery
SPR: surface plasmon resonance
TLR4: Toll-like receptor 4
TNFα: tumor necrosis factor-α
TUNEL: terminal deoxynucleotidyl transferase mediated dUTP nick-end labeling
Veh: vehicle
WT: wild-type.
