# Supplementary material for: Extracellular caspase-1: a critical inducer and a therapeutic target of lung injury in gut ischemia-reperfusion
Source: Front Immunol. 2026 Apr 15;17:1811868. doi: 10.3389/fimmu.2026.1811868 (PMC13124943; doi:10.3389/fimmu.2026.1811868)
Supplement: Supplementary file 1 [file DataSheet1.pdf]

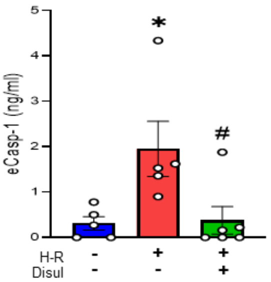

Supplemental Figure S1: eCasp1 is released from intestinal epithelial cells following hypoxia/reoxygenation, dependent on GSDMD-mediated membrane processes.
Primary mouse intestinal organoids were exposed to hypoxia for 6 h, followed by reoxygenation with or without Disulfiram (Disul.; 5 μM). After 16 h, culture supernatants were collected, and eCasp-1 was assessed by ELISA. Experiments were performed 3 times, and all data were used for analysis. Data were expressed as mean ± SEM (n = 5 samples/group) and compared by one-way analysis of variance and Student-Newman-Keuls method ($^{*}p < 0.05$ vs. PBS; $^{\#}p < 0.05$ vs. (+)H/R (-)Disul). Disul, Disulfiram.

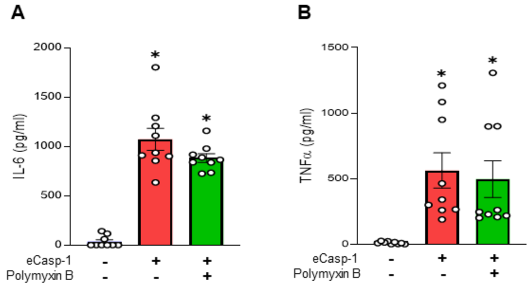

Supplemental Figure S2 : **Polymyxin B does not affect eCasp-1-induced cytokine production.**
(A, B) WT peritoneal macrophages were isolated and seeded into 96-well plates, then stimulated with PBS or eCasp-1 (0.1 μg/mL) for 4 h in the presence or absence of polymyxin B (15 μg/mL). Levels of (A) IL-6 and (B) TNFα in the culture supernatants were measured by ELISA. Experiments were performed 3 times, and all data were used for analysis. Data were expressed as mean ± SEM (n = 9 samples/group) and compared by one-way analysis of variance and Student-Newman-Keuls method ($^{*}p < 0.05$ vs. PBS).

Supplemental Table S1.

Primer sequences.

**Gene**	**Accession No.**	**Forward Primer**	**Reverse Primer**
IL-6	NM_031168	CCGGAGAGGAGACTTCACAG	CAGAATTGCCATTGCACAAC
TNFα	NM_013693	AGACCCTCACACTCAGATCATCTTC	TTGCTACGACGTGGGCTACA
IL-1β	NM_008361	CAGGATGAGGACATGAGCACC	CTCTGCAGACTCAAACTCCAC
KC	NM_008176	GCTGGGATTCACCTCAAGAA	ACAGGTGCCATCAGAGCAGT
MIP-2	NM_009140	CCCTGGTTCAGAAAATCATCCA	GCTCCTCCTTTCCAGGTCAGT
β-actin	NM_007393	CGTGAAAAGATGACCCAGATCA	TGGTACGACCAGAGGCATACAG
